# Registration of Births, Deaths and Marriages

**Published:** 1875-01

**Authors:** 


A Law for the Registration of Births, Deaths and Marriages may justly be regarded as one of the foot-prints of civilization. In calling attention to the subject at this time we do so not for the purpose of arguing the propriety of such a law, which would but insult the intelligence of our readers, but because the annual session of the Legislature of Georgia is near at hand, and we trust that this great public need will be pressed upon the attention of that body in such manner as to secure the passage of a wise and practicable law for the accomplishment of the end desired.

The whole subject was fully and ably discussed in our columns a year ago, and an earnest effort made during the legislative session to secure the law, but without success. There was, however, an interest awakened, and there is reason to believe that but for the want of sufficient unanimity of sentiment upon some minor points of details the measure would have passed. We hope that those of our readers who have influence with the powers that be will give the subject their attention, and aid in the passage of a suitable and efficient law for the accomplishment of ends so desirable and so necessary to the public weal.

Big Invention.—Lloyd, the famous map man, who made all the maps for General Grant, certificates of which he published, has just invented a way of getting a relief plate from steel, so as to print Lloyd's Map of the American Continent—showing from ocean to ocean—on one entire sheet of bank note paper,

40x50 inches large, on a lightning press, and colored, sized and varnished for the wall, so as to stand washing, and mailing anywhere in the world, for 25 cents, or unvarnished for 10 cents. This map shows the whole United States and Territories in a group, from surveys to 1875, with a million places on it, such as towns, cities, villages, mountains, lakes, rivers, streams, gold mines, railway stations, etc. This map should be in every house. Send 25 cents to the Lloyd Map Company, Philadelphia, and you will get a copy by return mail.

"The Galaxy" is about entering on its tenth year. It was started with the full intention of making it the foremost literary magazine published. Perfectly independent, with no set theories of politics, religion, or sociology to propagate and maintain, it freely and gladly opens its pages to the expression of varying opinions and discussions, provided they are by the ablest representatives in each department. All sides have a chance to speak through its pages on any subject which is exciting public interest and demands thoughtful discussion. That this plan has been a great success is proved by the brilliant history of this magazine for the past ten years. Our ablest statesmen and leading writers in all branches have expressed their views in its pages. During the coming year a series of articles is promised by prominent Southerners, giving the Confederate side of the war from its military and legislative standpoints. These articles will not be controversial, but will deal with facts to which both North and South will gladly give attention, as they will be written by men personally cognizant of what they speak. In the departments *The Galaxy* is especially rich. The "Scientific Miscellany" is particularly full and varied. The "Drift Wood," by Philip Quilibet, and the "Nebulæ," are very fresh and bright; and the department of "Current Literature" is well maintained. We heartily commend it to our readers.

Unusual Offer.—Dr. E. S. Gaillard, of Louisville, Ky., editor of the *Richmond and Louisville Medical Journal* and of the *American Medical Weekly*, makes to the public this unusual and liberal offer: To subscribers to the first journal, twelve handsomely engraved portraits of distinguished European and American physicians; to subscribers to the weekly, one of these portraits in each of the two volumes for 1875. The price of the first journal is $5 annually, and that of the last $2 for the same period.

One of our correspondents in Texas encloses the newspaper advertisement of an itinerant eye and ear doctor who advertises that "he has the endorsement of the medical profession of Waco," in that State. The advertisement has, to our *eye*, the distinctive *ear*-marks of quackery. It certainly cannot be defended under the code of ethics. Whatever may have been true in the above assertion prior to its publication in the Texas newspaper, we venture the confident opinion that the advertiser does not *now* bear the endorsement of the medical profession of Waco, or any other body of respectable medical men. Medical men of repute in the profession cannot be too careful in the bestowal of commendation and endorsement upon strangers.

We regret to learn that Dr. B. R. S. Boemond, of Jackson ville, Florida, was shot and killed, about the 15th of December, near Enterprise, in South Florida.

Recently, Dr. W. H. Hollingshead, of Fort Valley, Georgia, amputated the leg of Dr. J. L. Gibson, at Montezuma. The limb was crushed twenty years ago by an accident of some kind on a railroad. It was cut off below the knee.

